# *Fpemlocal*: Estimating family planning indicators in R for a single population of interest

**DOI:** 10.12688/gatesopenres.13211.1

**Published:** 2021-02-24

**Authors:** Gregory Guranich, Niamh Cahill, Leontine Alkema

**Affiliations:** 1 Department of Biostatistics and Epidemiology, University of Massachusetts Amherst, Amherst, USA; 2 Department of Mathematics and Statistics, Maynooth University, Kildare, Ireland

**Keywords:** Family Planning estimation tool, global versus local model fitting

## Abstract

The global Family Planning Estimation model (FPEM) combines a Bayesian hierarchical model with country-specific time trends to yield estimates of contraceptive prevalence and unmet need for family planning for countries worldwide. In this paper, we introduce the R package *fpemlocal* that carries out the estimation of family planning indicators for a single population, for example, for a single country or smaller area. In this implementation of FPEM, all non-population-specific parameters are fixed at outcomes obtained in a prior global FPEM run. The development of this model was motivated by the demand for computational efficiency, without loss of model accuracy, when estimates and projections from FPEM were needed only for a single country. We present use cases to produce estimates for a single population of women by union status or all women based on package-provided data bases and user-specified data. We also explain how to aggregate estimates across multiple populations. The R package forms the basis of the Track20 Family Planning Estimation Tool to monitor trends in family planning indicators for the FP2020 initiative. *Fpemlocal *is available from: https://github.com/AlkemaLab/fpemlocal

## Introduction

The global Family Planning Estimation model (FPEM) combines a Bayesian hierarchical model with country-specific time trends to yield estimates of contraceptive prevalence and unmet need for family planning. It was first designed to produce estimates for women aged 15–49 who are married or in a union, referred to here as in-union women, for 195 countries worldwide ( Alkema *et al.*, 2013; Cahill *et al.*, 2018). Subsequently, it has been used for producing estimates for women who are not in a union, referred to here as not_in_union women ( Kantorová *et al.*, 2020). The model accounts for differences by data source, sample population, and contraceptive methods included in the contraceptive use measure. The Bayesian hierarchical structure in the model is used to exchange information across countries regarding uptake of contraceptive methods, the relative share of modern versus traditional methods, and unmet need.

The local implementation of FPEM is a scaled-down version of the global FPEM, where the family planning (FP) model is fitted at a more local (or population-specific) level. Here we use the term local to refer to either in_union or not_in_union women in a single country or smaller area, referred to here as subdivisions. The distinction between the local FPEM and the global FPEM is that the local version can be run on data from a single population and in the model specification all non-population-specific parameters are fixed at outcomes obtained in the most up to date global FPEM run. The development of this model was originally motivated by the demand for computational efficiency, without loss of model accuracy, when estimates and projections from FPEM were needed only for a single country. Specifically, this local-run option was needed to facilitate the use of FPEM at country-support workshops run by the Track20 project ( *Track20*, n.d.). These workshops provided technical support to the pledging countries of the Family Planning 2020 initiative for monitoring progress in FP and the global FPEM was too computationally intensive to be useful. 

**Figure 1.**
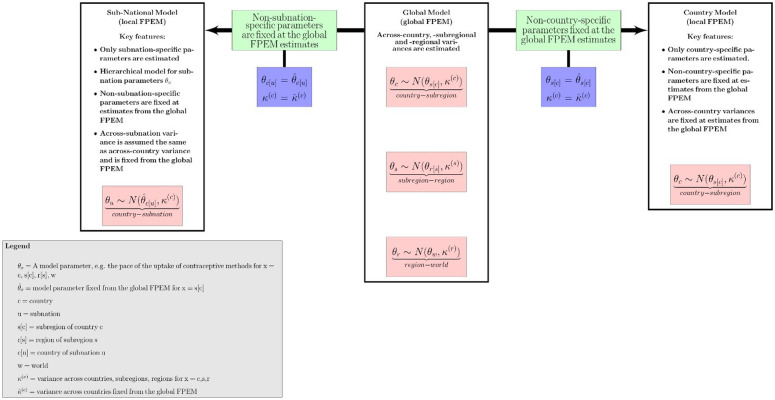
A flowchart to illustrate the relation between global and local FPEM. Figure taken from New *et al.* (2017), distributed with a CC BY license.

To illustrate how the local FPEM links to the global FPEM we have provided a flowchart in Figure 1 depicting (1) the hierarchical structure in the global FPEM and (2) the dependencies of the country and subnational local FPEM model specifications on the global FPEM output. In summary, in the country-specific implementation of the local FPEM non-country-specific parameters, e.g. the subregional pace of the uptake of contraceptive methods, across-country variances and the error variances and covariances for different data source types, were not estimated but were fixed at the point estimates obtained from a recent global model run. Similarly, FPEM can be fit to data from subdivisions, i.e. subnational populations. In the subnational implementation of the local FPEM, each subnation is considered as a “country” within the “subregion” of its respective nation. For instance, in India, States/UTs were considered as “countries” within the “subregion” of India. By fixing the subregional parameters at parameter point estimates obtained from the global run, this implementation of the local FPEM was used to obtain estimates of FP indicators for Indian states/UTs ( New *et al.*, 2017).

This paper introduces the R package *fpemlocal*. *fpemlocal* contains R functions and input data to do model fitting using the local implementation of FPEM. The package contains data from UNPD on contraceptive use and population numbers ( United Nations, Department of Economic and Social Affairs, Population Division, 2020a; United Nations, Department of Economic and Social Affairs, Population Division, 2020b). In addition, the package contains data from Track20 on contraceptive use. In this paper, we present different use cases for how to fit a model to data (UNPD or user-provided) to obtain estimates of contraceptive prevalence and users of contraceptive methods, among in_union or not_in_union women, for a country or subnational area. We also present how to obtain estimates that are aggregated across in_union and not_in_union women, and across geographical regions. 

## Methods

### Implementation

*fpemlocal* is an R package that contains R functions to carry out the pre- and postprocessing, and fit FPEM to data for a population of choice. Model fitting here refers to using Markov Chain Monte Carlo (MCMC) sampling to obtain samples from the posterior distributions of all model parameters.

### Operation

*fpemlocal* is a publicly available R package stored on Github. For usage, an R ( R Core Team, 2020) installation (≥3.4.0) and a JAGS ( Plummer, 2017) installation (≥4.0.0) are required. R can be downloaded on CRAN. JAGS is a program for the analysis of Bayesian models using MCMC. JAGS is written in C++ and is portable to all major operating systems. JAGS is available for download at https://sourceforge.net/projects/mcmc-jags/. Note that users will not interact with JAGS directly. Instead, *fpemlocal* will interface with JAGS through the dependency *R2jags.* ( Plummer, 2017; Su & Yajima, 2020)

*fpemlocal* can be installed with one of two methods: (1) direct install from Github repository using the *install_github()* API from the devtools package ( Wickham *et al.*, 2020); (2) install from the package binary using the base R function *install.package()*. Package dependencies are listed in the package DESCRIPTION file and will be automatically installed upon installing the main package. There are no minimum RAM, CPU, or HARDDRIVE requirements apart from what is necessary to store model runs, which varies case-by-case.

## Use cases

### Input data

The main functionality of *fpemlocal* is the fitting of the Bayesian FP estimation model to data for a population of interest. We describe its usage in the use cases in this section. Central to all examples are inputs in the form of a contraceptive use survey dataset (referred to as survey data), and a population count dataset (referred to as population data), for the population of interest. *fpemlocal* contains survey data and population data provided by the UNPD ( United Nations, Department of Economic and Social Affairs, Population Division, 2020a, United Nations, Department of Economic and Social Affairs, Population Division, 2020b). Help files provide the metadata related to these data sets, e.g. *?contraceptive_use* will display the helpfile for the contraceptive use survey dataset metadata, see ‘package data’ vignette for details. In summary, the contraceptive use survey dataset includes family planning data by, location, age, and marital status. Data are in the form of aggregated survey responses, i.e., prevalence, their transformations, and sampling errors where available. The function *impute_user_se* is used for imputing missing sampling errors in custom survey data prior to model fitting, based on the approach outlined in Cahill *et al.* (2018) (appendix p. 16). The population count dataset includes population counts by year, location, age, and marital status. *Fpemlocal* supports the use of external datasets in place of the default package datasets as long as the format follows that of the default data sets (see use case 1.2). 

Additional data provided with the package is used for model fitting and consists of (1) parameter estimates to use in local FPEM, and (2) information on how countries are organized in hierarchical groupings. Parameter estimates are obtained from the most recent UNPD global FPEM runs, currently those of the 2020 revision ( United Nations, Department of Economic and Social Affairs, Population Division, 2020b). Information on hierarchical groupings (geographical or otherwise) is provided in the dataset *divisions*.

### Model fitting

The primary function of *fpemlocal*, *fit_fp_c*, fits the family planning model for a geographical population of interest, for in_union women and/or not_in_union women. Its arguments are summarized in Table 1. The first and primary argument to *fit_fp_c*, is a contraceptive use survey dataset. If the user does not supply a survey dataset, the package survey dataset is used. Subsequent arguments *division_numeric_code* and *is_in_union* are used to filter input data to obtain the inputs and parameter values that are relevant to the population of interest. In particular, the division code and union status will determine the groupings applied when fitting the local FPEM, and the selected survey observations. Argument *is_in_union* determines if results are to be obtained for in_union, not_in_union or all women. If results are to be obtained for all women, model fits for in_union and not_in_union women are obtained.

**Table 1.** Argument descriptions for function *fit_fp_c.*

Argument	Data type	Description
surveydata_filepath	Character	Path to survey data. Survey data should be a .csv. When left NULL, the function will default to the package dataset `contraceptive_use`
division_numeric_code	Numeric	A numeric code associated with the country. This code will determine the groupings applied when fitting FPEM. See the data from `divisions`
is_in_union	Character	Specify the union status of women. Options are in-union, not-in-union, and all women. “Y”, “N”, and “ALL” respectively. Default is “Y”.
first_year	Numeric	The first year of model estimates returned. The model will be fit to all data, including dates before this date if available.
last_year	Numeric	The last year of model estimates returned. The model will be fit to all data, including dates after this date if available.

### Model output

After calling *fit_fp_c*, the resulting fit object contains posterior samples of all relevant model parameters and essential fit data called core data. Core data is a list containing processed survey data, bias-adjusted survey data, other *fit_fp_c* inputs, and information about the model fit.

Model output is supplied to *calc_fp_c* where quantiles of posterior samples are calculated to produce credible intervals. The estimates from *calc_fp_c* in conjunction with the fit object returned from *fit_fp_c* can be supplied to *plot_fp_c* to generate plots of estimates over time overlapping input data.

### Case 1: Estimating FP indicators for in_union or not_in_union women

**Figure 2.**
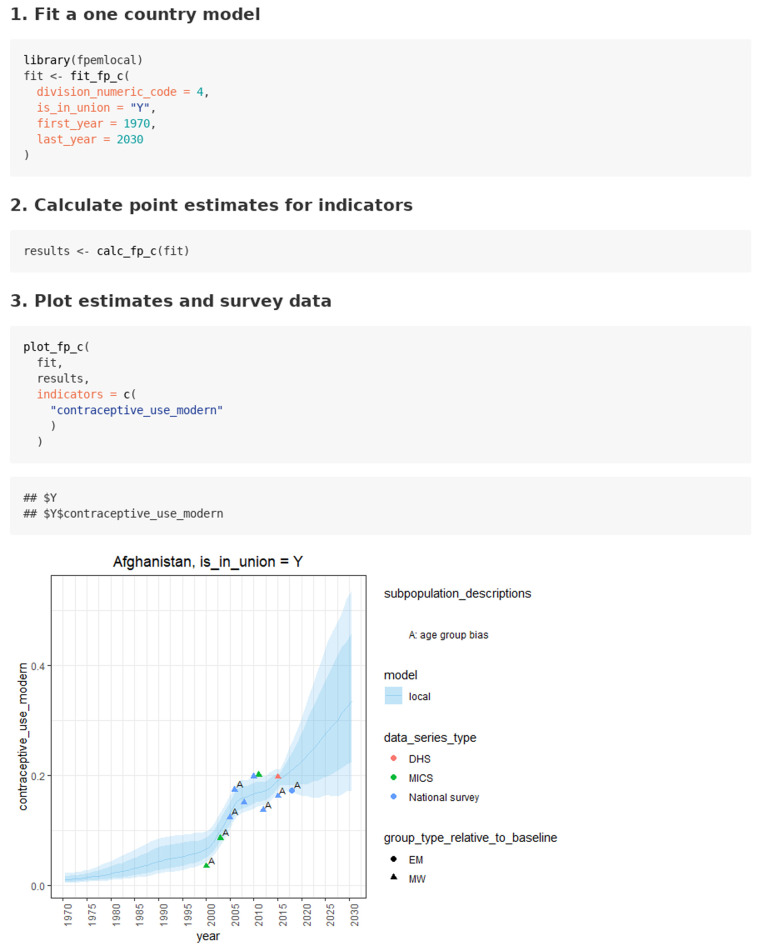
Function calls for use case 1.1, estimating family planning indicators for in_union or not_in_union women for Afghanistan (country numeric code 4) with an illustration of a plot for modern contraceptive prevalence estimates over time from the function *plot_fp_c*. Results are shown for Afghanistan. Light purple shaded area represents 95% credible intervals and the dark purple area represents 80% credible intervals.

***Case 1.1: (default case) FP estimation with UNPD package datasets.*** The use case of estimating FP indicators for a country of interest using default UNPD data is given in the ‘in union women’ vignette. This vignette takes less than one minute to run on a machine with an 8 core 3.60GHz CPU and 16GB of RAM. In this use case, the user starts by finding the country code (division numeric code) for the country of interest. Then, the user calls the function *fit_fp_c* to fit the local FPEM model. The user supplies the country code, the union status, and the years of estimates to be returned (see Figure 2). The user does not supply survey data. By default, the function loads package survey data. The function *fit_fp_c* returns posterior samples and core data. After the model is fit, the user calls the function *calc_fp_c* to calculate family planning indicators. The calculation of some indicators requires population counts. By default, the function *calc_fp_c* loads package population count data if it is not supplied by the user. Lastly, the user supplies the fit object, the results object, and a vector of indicator names to the function *plot_fp_c* to plot indicator estimates and survey data (see Figure 2).

***Case 1.2: Estimating FP indicators using custom datasets.*** The use case for FP estimating using custom datasets is given in the ‘in union women from custom data’ vignette. Similar to case 1.1, the user starts by fitting the model with the function *fit_fp_c*. In addition to the inputs in case 1.1, the user supplies the file path of the .csv file containing the survey dataset. Any missing sampling errors in the input data are imputed automatically with the function *input_user_se*.

**Figure 3.**
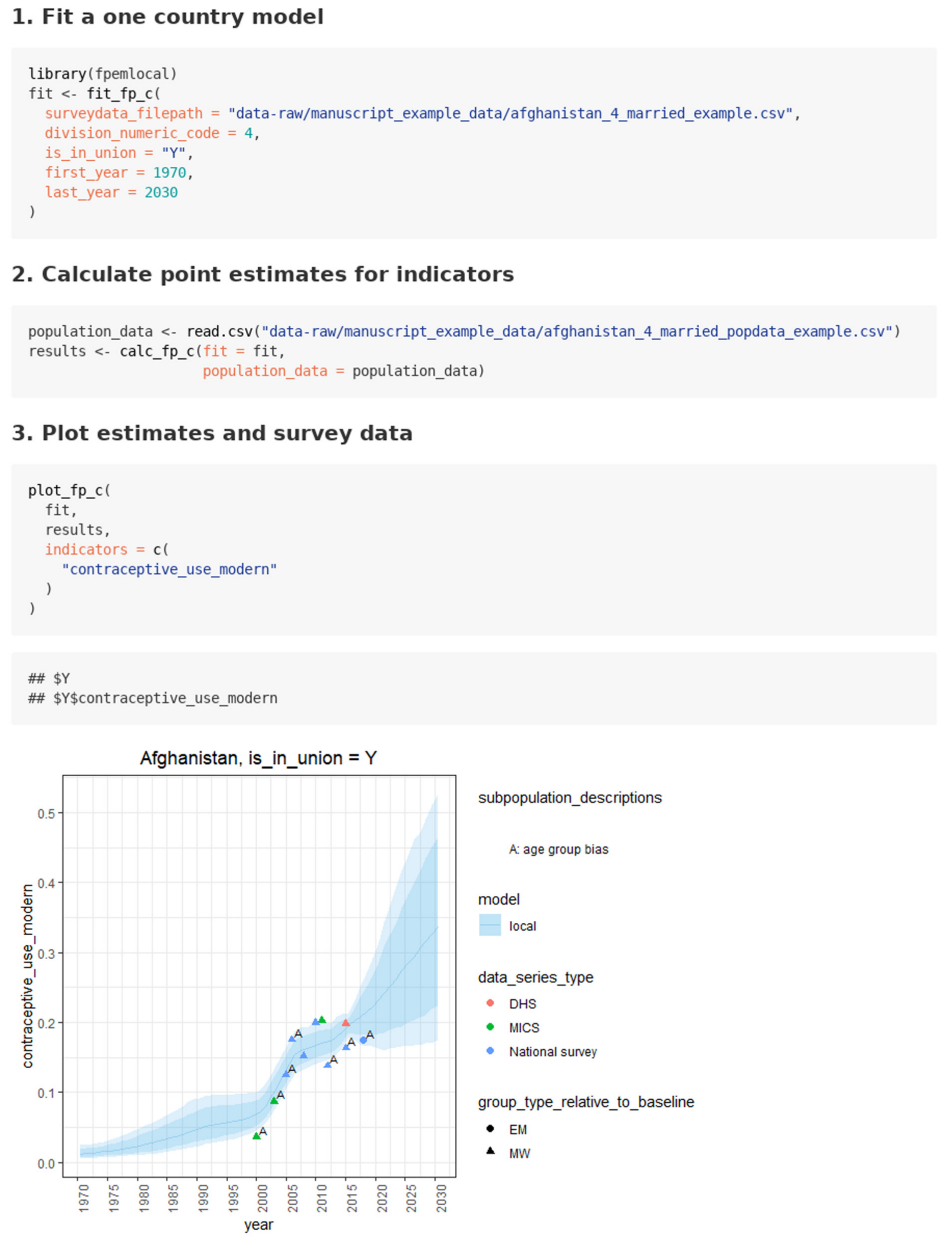
Function calls and illustrative output for use case 1.2: Estimating FP indicators for married women with custom user data.

After the model is fit, the user reads in the .csv file containing the population count data for the population of interest (see Figure 3). Next, they supply the fit object and the population count data to the function *calc_fp_c*. Results can be plotted using the *plot_fp_c* function.

***Case 1.3 Estimating FP indicators with custom subnational datasets.*** The user must supply a custom dataset for the use of *fpemlocal* for subnational estimation, as no default datasets are available. The use case for subnational estimation with custom datasets is given in the ‘subnational’ vignette. The use case follows that from case 1.2 with the only change being that the user sets the argument *subnational* equal to *TRUE* when calling subnational *fit_fp_c*. Internally, this results in using parameters for model fitting that are relevant for subnational runs, considering each subnation to be a “country” within the “subregion” of its respective nation. For example, mean pace of the uptake parameters are obtained from the country of interest (as indicated by *division code*) as opposed to its larger region. As in case 1.2, the user supplies custom survey data for model fitting and custom population counts.

### Case 2: Estimating FP indicators for all women

**Figure 4.**
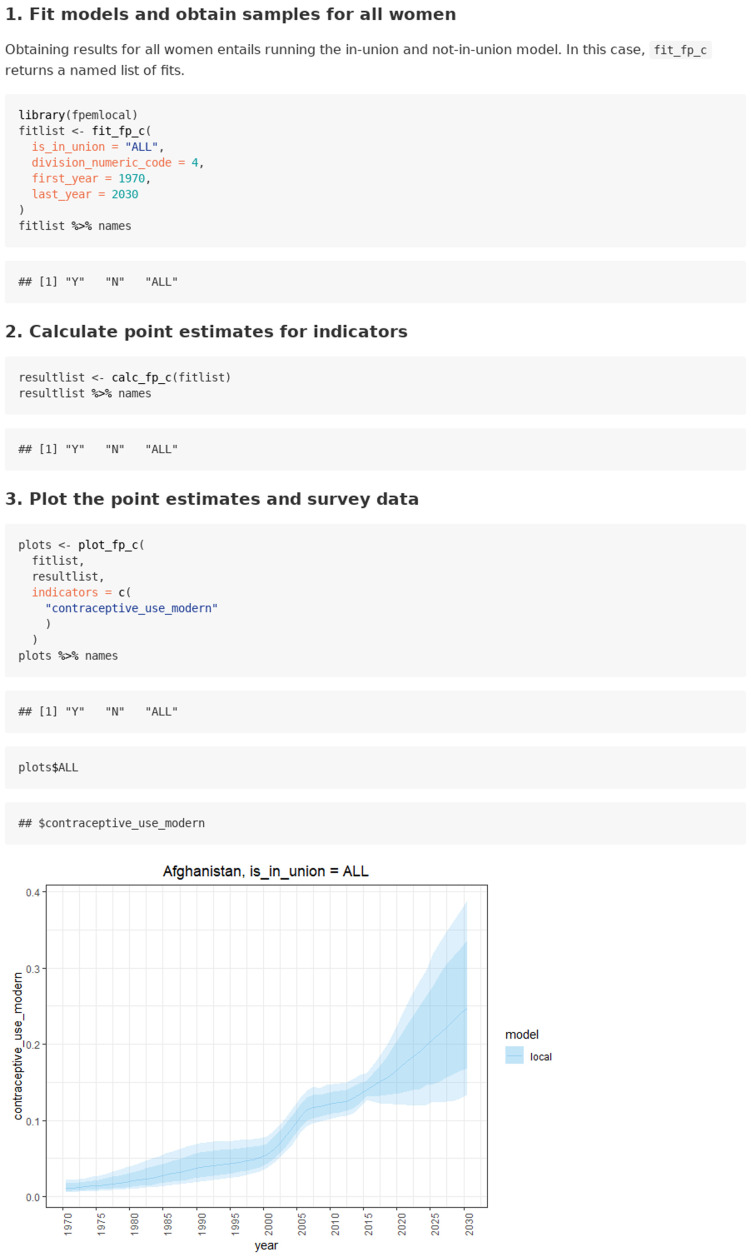
Function calls and illustrative output for use case 2: Estimating FP indicators for all women.

The use case for estimating FP indicators for all women is given in the `estimating for all women` vignette. Obtaining results for both in_union and not_in_union women entails running the in-union and not-in-union model. In this use case, the user can supply survey data for in_union and not_in_union women or use the default UNPD data base, and the model is fit to both by specifying argument *is_in_union = “ALL”* in the model fit function call. The resulting fit object is a named list of fits (see Figure 4).

Next, the user supplies the entire list of fits from *fit_fp_c* to the function *calc_fp_c*. Like the previous function, *calc_fp_c* returns a list of results with estimates for in-union women, not-in-union women, and all women. Results can be plotted using the *plot_fp_c* function.

### Case 3: Aggregating multiple fits and obtaining aggregate estimates

*fpemlocal* allows users to aggregate estimates from multiple populations to produce estimates that refer to the combined population. Aggregate estimates of family planning proportions - referring to contraceptive use, unmet need, and the no need category - are given by the weighted average of population-specific outcomes, with weights given by the number of women in the respective population. For example, to obtain modern use among in_union women, the weights are given by the number of in_union women in each single population that is combined in the aggregate outcome.

**Figure 5.**
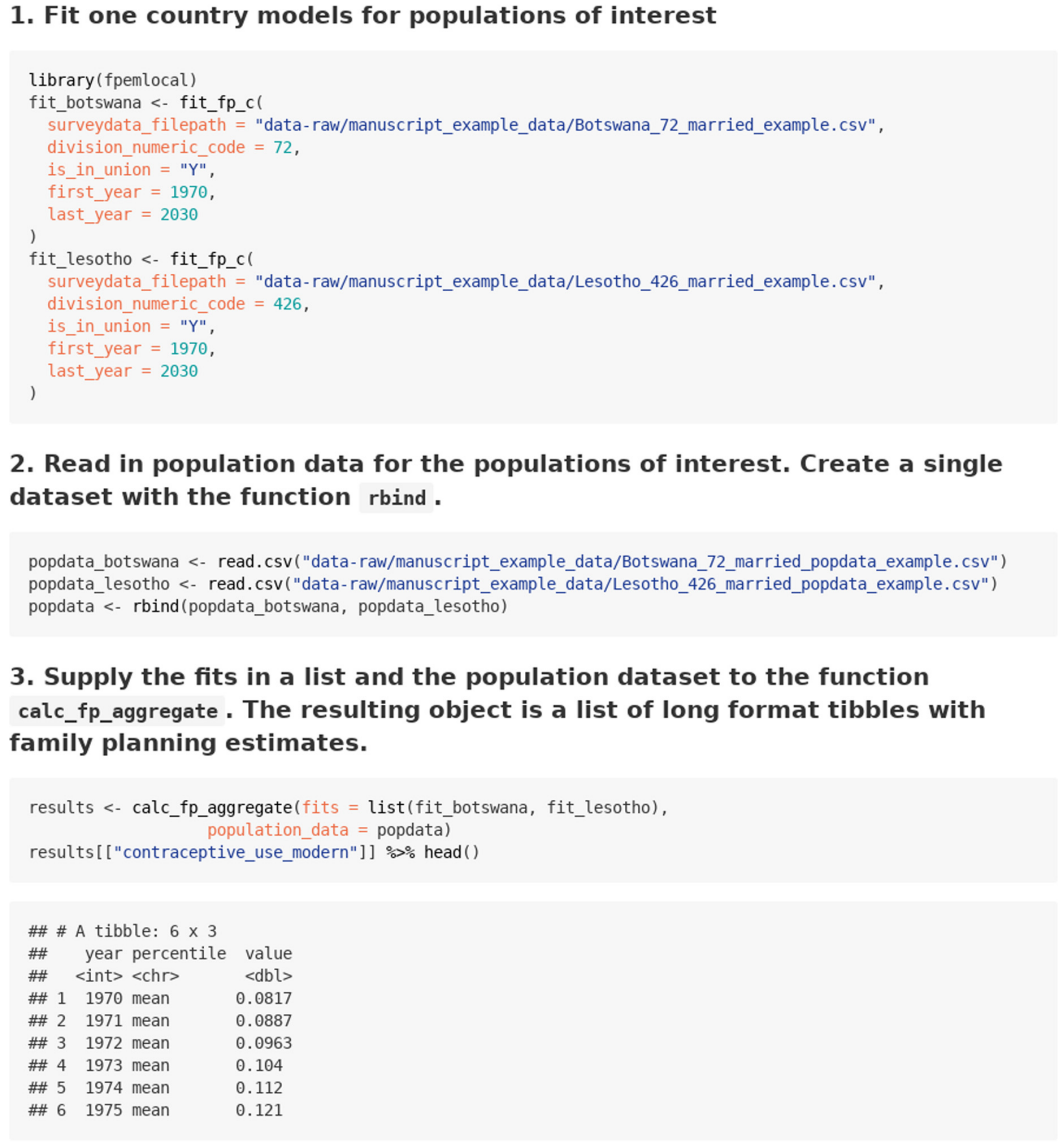
Function calls and illustrative output for use case 3: Aggregating multiple fits and obtaining aggregate estimates.

The case for aggregating multiple fits is given in the ‘aggregating estimates’ vignette and summarized in Figure 5. First, the user fits FPEM to each population of interest. Next, the user prepares a single population count dataset containing all populations of interest. The user supplies the fit objects and the population data to the function *calc_fp_aggregate* to obtain aggregate estimates.

### Additional features

*Automatic fit saving: fpemlocal* includes wrapper functions, built around the functions described so far, to automatically save outputs. These functions may be useful to users fitting the model to multiple countries. The wrapper functions are given by *fit_fp_c_autosave*, *calc_fp_cautosave*, and *plot_fp_c_autosave*.

*Diagnostics:* The fit function *fit_fp_c* includes an option to save diagnostic checks in the form of trace plots and convergence checks ( Vehtari *et al.*, 2020). Default settings are used in model fitting based on analysis of these diagnostics.

*Service statistics data*: Service statistics data as summarized into Estimated Modern Use (EMU) can be included in the model fitting as well ( Magnani *et al.*, 2018).

## Conclusions

We introduced the R package *fpemlocal*. This package can be used to fit the local Family Planning Estimation Model to populations of interest. The package is used by Track20 for use in country workshops, through an online interface ( Track20, n.d.) which has informed FP2020 initiative reporting since 2013. The package is used by Track20 which has informed FP2020 initiative progress reports ( FP2020, 2019). Recent additional use of *fpemlocal* includes the assessment of the increase in modern contraceptive use needed to reach demand satisfied targets by 2030 ( Cahill *et al.*, 2020).

*Fpemlocal* can serve as an example for other global modeling exercises. Publishing the code in the form of an R package facilitates the production, reproduction, and transparency, of model-based estimates. The local implementation of a global model makes fitting less computationally demanding, thus enabling users with limited computational resources to fit Bayesian models to populations of their choice, as indicated in the case studies.

## Software availability

Software source code: https://github.com/AlkemaLab/fpemlocal

Archived source code as at time of publication: https://doi.org/10.5281/zenodo.4302624 ( Guranich *et al.*, 2020).

License: MIT license
