# Supplementary material for: Fpemlocal: Estimating family planning indicators in R for a single population of interest
Source: Gates Open Res. 2021 Feb 24;5:24. [Version 1] doi: 10.12688/gatesopenres.13211.1 (PMC8008158; doi:10.12688/gatesopenres.13211.1)
Supplement: Peer review history

# Peer review: Reviewer response for version 1

**DOI:** 10.21956/gatesopenres.14429.r30447

**Published:** 2021-03-29

**Authors:** Qingfeng Li

**Affiliations:** 1 Department of International Health, Johns Hopkins Bloomberg School of Public Health, Baltimore, MD, USA

The authors present an R package named fpemlocal, a local implementation of a previously published global Bayesian hierarchical model. Similar functions are already available through an online version, but this R package may be more convenient for users who prefer the R interface and have unstable internet access. With the instructions from the article and README on GitHub, the package is user-friendly overall. Some suggestions for further improvement:

1. For use case 1.1, perhaps the package can directly include the results from a global run. The package only needs to extract the results for the country of interest to the user, instead of fitting a single-country model for the given country. Theoretically, by fixing non-country-specific parameters at the global FPEM estimates, the single-country implementation compromises the model accuracy. For use cases 1.2 and 1.3, the substantial saving in computational load overwhelms the slight loss in accuracy. But that does not apply to case 1.1.
2. Customize figure appearance. Current output figures are nice and clear, but they require some final minor changes if the user wants to include them in a presentation or a report. For example, replace variable names on axis titles with properly capitalized words. An easy way to achieve that is by changing the default setting and adding those arguments to the plot_fp_c function.
3. The users may need more instructions on preparing data for cases 1.2 and 1.3. The instructions should include column definitions and clarify which columns are mandatory. 

Are the conclusions about the tool and its performance adequately supported by the findings presented in the article?

Yes

Is the rationale for developing the new software tool clearly explained?

Yes

Is the description of the software tool technically sound?

Yes

Are sufficient details of the code, methods and analysis (if applicable) provided to allow replication of the software development and its use by others?

Yes

Is sufficient information provided to allow interpretation of the expected output datasets and any results generated using the tool?

Yes

Reviewer Expertise:

demographic and statistical modeling; public health

I confirm that I have read this submission and believe that I have an appropriate level of expertise to confirm that it is of an acceptable scientific standard.

# Peer review: Reviewer response for version 1

**DOI:** 10.21956/gatesopenres.14429.r30449

**Published:** 2021-03-04

**Authors:** Oliver Stevens

**Affiliations:** 1 Department for Infectious Disease Epidemiology, School of Public Health, Imperial College London, London, UK

This article reports the implementation and use of the *fpemlocal *package, a slimmed down version of FPEM for estimating family planning indicators at the single country level. Both the manuscript and the package itself are clearly laid out and easy to follow. The model fits quickly on a domestic laptop and performs well against survey data tested.

 Fixing the model hyperparameters to global posterior point estimates is a good solution to reduce computational burden. It may be useful to country team users to include the globally fitted FPEM results as a *fpemlocal *package dataset with which *fpemlocal* results may be overlaid.

 Some minor comments:

- Functions would benefit from improved help text, including examples with working arguments. Without the examples in the manuscript which future users may not consult, it was not immediately clear how functions should be called.

fit_fp_c()

- It would be useful to state which arguments for fit_fp_c() are mandatory and which are optional/autofilled by the package.
- Following fitting, all the model internals are placed individually into the global environment. Please nest as a list within the fit object.

plot_fp_c()

- plot_fp_c() requires ggnewscale as a dependency which is not installed with the package.
- Help text for plot_fp_c argument "results" *Results data from fpem_calculate_results*, but should read "results data from calc_fp_c()"
- Plots from plot_fp_c():
  - Axes and legend labels should be more human readable - e.g. "Modern Contraceptive Use" vs "contraceptive_use_modern"
  - Please display confidence intervals on survey estimates
  - Not clear what group_type_relative_to_baseline represents

Future work may look to implement a spatial model when single country fits are done at the subnational level - beyond the scope of this software report.

 The subnational vignette, though setting subnational = TRUE, doesn't use subnational data & accordingly doesn't generate subnational results, so it's difficult to comment on its usage.

Are the conclusions about the tool and its performance adequately supported by the findings presented in the article?

Yes

Is the rationale for developing the new software tool clearly explained?

Yes

Is the description of the software tool technically sound?

Yes

Are sufficient details of the code, methods and analysis (if applicable) provided to allow replication of the software development and its use by others?

Yes

Is sufficient information provided to allow interpretation of the expected output datasets and any results generated using the tool?

Yes

Reviewer Expertise:

Infectious disease epidemiology/demography

I confirm that I have read this submission and believe that I have an appropriate level of expertise to confirm that it is of an acceptable scientific standard.
